# The impact of higher protein dosing on outcomes in critically ill patients with acute kidney injury: a post hoc analysis of the EFFORT protein trial

**DOI:** 10.1186/s13054-023-04663-8

**Published:** 2023-10-18

**Authors:** Christian Stoppe, Jayshil J. Patel, Alex Zarbock, Zheng-Yii Lee, Todd W. Rice, Bruno Mafrici, Rebecca Wehner, Man Hung Manuel Chan, Peter Chi Keung Lai, Kristen MacEachern, Pavlos Myrianthefs, Evdoxia Tsigou, Luis Ortiz-Reyes, Xuran Jiang, Andrew G. Day, M. Shahnaz Hasan, Patrick Meybohm, Lu Ke, Daren K. Heyland

**Affiliations:** 1 University Hospital Würzburg, Department of Anaesthesiology, Intensive Care, Emergency and Pain Medicine, Oberdürrbacher Str. 6, 97080 Würzburg, Germany; 2 Department of Cardiac Anesthesiology and Intensive Care Medicine, Charité Berlin, Berlin, Germany; 3 Division of Pulmonary and Critical Care Medicine, Medical College of Wisconsin, Milwaukee, WI USA; 4 Department of Anaesthesiology and Intensive Care Medicine, University of Münster, Münster, Germany; 5 Department of Anaesthesiology, Faculty of Medicine, University of Malaya, Kuala Lumpur, Malaysia; 6 Division of Allergy, Pulmonary, and Critical Care Medicine, Vanderbilt University Medical Center, Nashville, TN USA; 7 Renal and Transplantation Unit, Department of Dietetics and Nutrition, Nottingham University Hospitals NHS Trust, City Campus, Nottingham, UK; 8 Department of Clinical Nutrition, The Ohio State University Wexner Medical Center, Columbus, OH USA; 9 Department of Adult Intensive Care, Queen Mary Hospital, Hong Kong, China; 10 Intensive Care Unit, Mount Sinai Hospital, Sinai Health System, Toronto, Canada; 11 Intensive Care Unit, Agioi Anargiroi Hospital, Athens, Greece; 12 Clinical Evaluation Research Unit, Queen’s University, Watkins 5, Kingston General Hospital, Kingston, ON K7L 2V7 Canada; 13 Department of Critical Care Medicine, Queen’s University, Watkins 5, Kingston General Hospital, Kingston, ON K7L 2V7 Canada; 14 Department of Critical Care Medicine, Jinling Hospital, Medical School of Nanjing University, Nanjing, China

**Keywords:** Acute kidney injury, Critical illness, Nutrition support, Protein, Randomized trial, Registry trial

## Abstract

**Background:**

Based on low-quality evidence, current nutrition guidelines recommend the delivery of high-dose protein in critically ill patients. The EFFORT Protein trial showed that higher protein dose is not associated with improved outcomes, whereas the effects in critically ill patients who developed acute kidney injury (AKI) need further evaluation. The overall aim is to evaluate the effects of high-dose protein in critically ill patients who developed different stages of AKI.

**Methods:**

In this post hoc analysis of the EFFORT Protein trial, we investigated the effect of high versus usual protein dose (≥ 2.2 vs. ≤ 1.2 g/kg body weight/day) on time-to-discharge alive from the hospital (TTDA) and 60-day mortality and in different subgroups in critically ill patients with AKI as defined by the Kidney Disease Improving Global Outcomes (KDIGO) criteria within 7 days of ICU admission. The associations of protein dose with incidence and duration of kidney replacement therapy (KRT) were also investigated.

**Results:**

Of the 1329 randomized patients, 312 developed AKI and were included in this analysis (163 in the high and 149 in the usual protein dose group). High protein was associated with a slower time-to-discharge alive from the hospital (TTDA) (hazard ratio 0.5, 95% CI 0.4–0.8) and higher 60-day mortality (relative risk 1.4 (95% CI 1.1–1.8). Effect modification was not statistically significant for any subgroup, and no subgroups suggested a beneficial effect of higher protein, although the harmful effect of higher protein target appeared to disappear in patients who received kidney replacement therapy (KRT). Protein dose was not significantly associated with the incidence of AKI and KRT or duration of KRT.

**Conclusions:**

In critically ill patients with AKI, high protein may be associated with worse outcomes in all AKI stages. Recommendation of higher protein dosing in AKI patients should be carefully re-evaluated to avoid potential harmful effects especially in patients who were not treated with KRT.

*Trial registration*: This study is registered at ClinicalTrials.gov (NCT03160547) on May 17th 2017.

**Supplementary Information:**

The online version contains supplementary material available at 10.1186/s13054-023-04663-8.

## Background

Critical illness is frequently accompanied by acute kidney injury (AKI) [1–4]. AKI impairs homeostatic functions, including the maintenance of acid–base balance and resultant metabolic acidosis, which further increases proteolysis, protein catabolism and impairs transcellular amino acid transportation [5]. This loss of renal metabolic function impairs amino acid conversions and utilization [6]. In severe AKI, the use of kidney replacement therapy (KRT) further exacerbates amino acid loss [7]. Moreover, critical illness defining conditions such as sepsis, respiratory failure, and trauma lead to proteolysis with a negative nitrogen balance and an acquired loss of muscle mass, which impairs physical functioning, recovery and ultimately quality of life in survivors [8, 9]. Therefore, in theory, critically ill patients with AKI may require a greater protein dose, compared to patients without AKI.

This is reflected in the current nutrition guidelines recommend targeting a protein dose up to 2.0 g/kg body weight (BW)/d for patients with AKI not on KRT and up to 2.5 g/kg BW/d for patients with AKI on KRT [10, 11]. The recently completed EFFORT Protein trial among mechanically ventilated critically ill patients demonstrated that higher (≥ 2.2 g/kg/BW/d), compared to usual (< 1.2 g/kg/BW/d) protein dose, did not improve time-to-discharge-alive from hospital (TTDA) nor 60-day mortality. However, in an a priori defined subgroup of patients with AKI, high-dose protein, compared to lower, was associated with worse outcomes [12]. Therefore, we performed this secondary analysis of the EFFORT protein trial to further explore the impact of protein dose among different subgroup of patients with AKI. Among these, the influence of protein in the different stages of AKI, the use and duration of KRT post randomization, and potential influence of chronic kidney disease (CKD) at baseline were of special interest. We hypothesize that compared to a lower dosing, higher protein dosing is associated with worse clinical outcomes in critically ill patients with AKI at any stage who were not treated with KRT.

## Methods

### Study design and participants

We conducted an exploratory secondary analysis of the EFFORT Protein trial, an international, prospective, investigator-initiated, pragmatic, registry-based, randomized, single-blinded trial, in 85 Intensive Care Units (ICUs) across 15 countries.

The primary objective of this trial was to evaluate if delivering a higher, compared to usual protein dose, in mechanically ventilated critically ill patients with high nutritional risk would result in reduced TTDA. Briefly, this study enrolled adult (age ≥ 18) patients with one of the following nutrition risk factors within 96 h of ICU admission: body mass index (BMI) ≤ 25 or ≥ 35 kg/m^2^, [13] moderate to severe malnutrition as defined by local assessment, clinical frailty scale ≥ 5 [14], SARC-F ≥ 4 [15], or projected duration of mechanical ventilation for > 4 days. Patients were excluded if they were moribund, pregnant, or when equipoise of protein dose was not present [12]. In this secondary analysis, we included a subset of patients with AKI, which is defined by the Kidney Disease Improving Global Outcomes (KDIGO) classification within the first 7 days after ICU admission using serum creatinine. [16]

### Intervention

A detailed description of the protein intervention can be found in the primary publication [12]. Briefly, using concealed allocation, eligible patients were randomly assigned using random-sized permuted blocks stratified by ICU to receive a high (≥ 2.2 g/kg/BW/d) or usual (≤ 1.2 g/kg/BW/d) protein target as soon as possible after randomization, and continued for up to 28 days in the ICU, before the transition to full and permanent oral feeding. Nutrition targets for calories and proteins were set using pre-ICU actual dry weight, whereas for patients with BMI > 30 kg/m^2^, an ideal body weight, based on a BMI of 25 kg/m^2^, was used. In this pragmatic trial, the energy dose was determined by the primary clinical team; however, we discouraged overfeeding.

### Outcomes of this secondary analysis

The primary outcome for this secondary analysis was to evaluate the impact of protein dose on TTDA among patients with AKI. Death was a competing risk and patients who died within 60 days of ICU admission were considered to have never been discharged alive regardless of prior hospital discharge. Secondary outcomes included 60-day mortality, duration of KRT from randomization, and incidence of KRT post randomization. In addition, we also compared the urea levels between groups during the first 12 study days. Clinical outcomes were assessed up to a maximum of 60 days post randomization while the patient was in the hospital. Protein and energy delivery were assessed for the first 28- and 12-days post randomization, respectively.

### Statistical analysis

In view of the persistent COVID-19 pandemic, enrolment rates in this volunteer-driven trial significantly decreased and achieving the original sample size was not feasible. Based on the collected data and event rates at that time, a sample size recalculation was performed and the Steering Committee decided to switch the primary outcome from 60-day mortality to time-to-discharge-alive from hospital [12]. We described the impact of protein dose assignment on TTDA by the substitution hazard ratio estimated by extending the Cox proportional hazards model to allow for death as a competing risk [17] and ICU as a shared frailty (i.e. random effect) [18]. Since SAS does not implement both the Fine-Gray approach and shared frailty model simultaneously, we approximated the Fine-Gray estimates by censoring deaths after the last event time. We confirmed that in the absence of a random ICU effect, our approximation would provide identical results to the true Fine-Gray estimator to the decimals reported. For 60-day mortality, we report relative risks estimated by the mixed log-binomial model with ICU as a random effect using maximum likelihood estimation based on Laplace approximation.

*p*-values for other variables compared between treatment groups were estimated by the chi-squared test for categorical variables and the Mann–Whitney U test for numeric variables. We used all available data without imputation due to the small amount of missing data; we report the number of patients used throughout the analysis.

### Effect modification analysis

We hypothesized that among patients with AKI, the following subgroups may modify the association of protein dose on TTDA or 60-day mortality: AKI stage 1, 2 or 3, received vs not received KRT post-randomization (regardless of KRT prior to randomization), ever vs never dialyzed prior to or during the current ICU stay, CKD versus no CKD, blood urea nitrogen to creatinine ratio (BUN; > or ≤ 22), age > or ≤ 59, BMI > or ≤ 30, mNUTRIC ≥ or < 5, SOFA ≥ or < 9, APACHE II ≥ or < 21, medical vs surgical patients, sepsis vs non-sepsis patients, patients who were in shock vs not in shock, frailty ≥ or < 5, SARC-F ≥ or < 4, malnourished vs non-malnourished. The within subgroup estimates and tests for subgroup by treatment interaction were obtained by adding a treatment arm by subgroup interaction term to the models previously described to analyze TTDA and 60-day mortality. We tested for interactions between protein target and the 16 subgroups across two outcomes, but since no test for interaction reached statistical significance at 0.05, we did not adjust *p*-values for multiplicity. The analysis was performed using SAS Version 9.4 (SAS Institute Inc., Cary, NC, USA).

## Results

### Patients’ characteristics

**Fig. 1:**
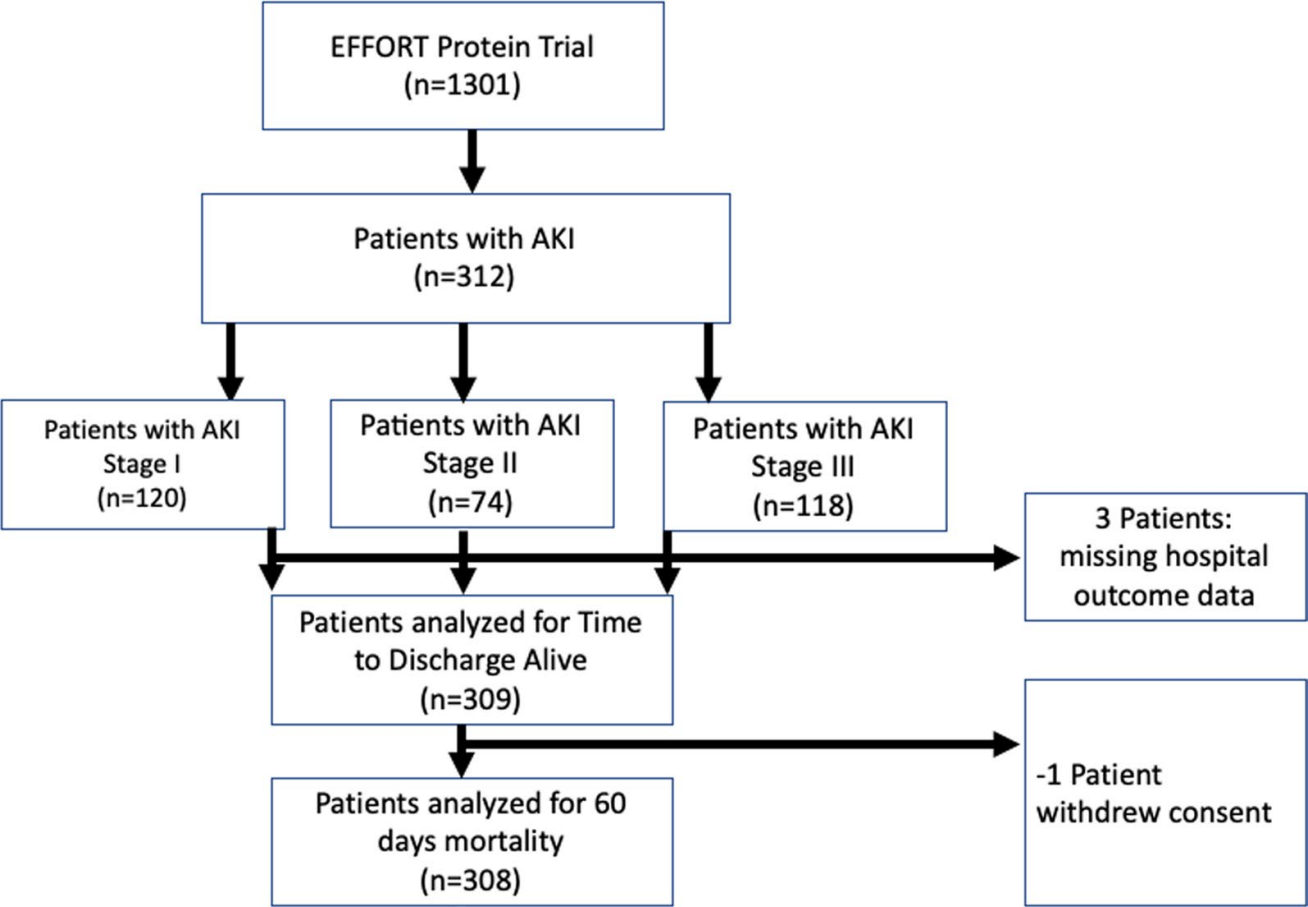
Flow chart

In the main trial, 1329 patients were randomized: 645 to receive high-dose protein and 656 to receive usual-dose protein. In the high-protein group, 163 (25.3%) patients had AKI while in the usual care group, 149 (22.7%) had AKI. In total, 312 patients developed AKI within the first 7 days in the ICU were included in this analysis, whereby 120 (38.5%), 74 (23.7%), 118 (37.8%) were AKI stage I, II and III, respectively (Fig. 1). Baseline characteristics were similar between the groups (Table 1) as were the number of patients who had CKD (21.5% vs. 18.5%) or received new or ongoing KRT after randomization (36.8% vs. 31.5%).

**Table 1:** Baseline demographics in AKI patients

Characteristic	All patients	High protein	Usual protein
*N*	312	163	149
Age (years)	(312) 60.7 ± 14.6 (20.0–95.0)	(163) 60.8 ± 13.6 (22.0–95.0)	(149) 60.6 ± 15.7 (20.0–93.0)
*Sex*			
Male	191 (61.2%)	102 (62.6%)	89 (59.7%)
Female	120 (38.5%)	61 (37.4%)	59 (39.6%)
*Admission status*			
Medical	278 (89.1%)	144 (88.3%)	134 (89.9%)
Surgical elective	11 (3.5%)	5 (3.1%)	6 (4.0%)
Surgical emergency	23 (7.4%)	14 (8.6%)	9 (6.0%)
*ICU admission status*			
Baseline SOFA score	(312) 9.7 ± 3.7 (0.0–22.0)	(163) 9.7 ± 3.5 (0.0–18.0)	(149) 9.7 ± 3.9 (0.0–22.0)
APACHE II score	(298) 23.7 ± 8.0 (6.0–55.0)	(155) 23.7 ± 7.6 (6.0–45.0)	(143) 23.7 ± 8.3 (6.0–55.0)
NUTRIC score	(298) 5.4 ± 1.7 (0.0–9.0)	(155) 5.3 ± 1.6 (0.0–9.0)	(143) 5.4 ± 1.9 (1.0–9.0)
Frailty	(284) 3.9 ± 1.6 (1.0–8.0)	(152) 3.9 ± 1.6 (1.0–8.0)	(132) 3.9 ± 1.7 (1.0–8.0)
Frailty (clinical frailty scale ≥ 5)	94 (30.1%)	48 (29.4%)	46 (30.9%)
*Patients received renal replacement therapies on randomization day*			
Yes	69 (22.1%)	39 (23.9%)	30 (20.1%)
No	243 (77.9%)	124 (76.1%)	119 (79.9%)
*Patients ever received KRT post randomization*			
Yes	107 (34.3%)	60 (36.8%)	47 (31.5%)
No	205 (65.7%)	103 (63.2%)	102 (68.5%)
*Patients ever versus never dialyzed*			
Yes	116 (37.2%)	65 (39.9%)	51 (34.2%)
No	196 (62.8%)	98 (60.1%)	98 (65.8%)
Acute kidney injury*			
Stage 1	120 (38.5%)	59 (36.2%)	61 (40.9%)
Stage 2	74 (23.7%)	43 (26.4%)	31 (20.8%)
Stage 3	118 (37.8%)	61 (37.4%)	57 (38.3%)
*Chronic kidney disease*			
Yes	63 (20.2%)	35 (21.5%)	28 (18.8%)
No	249 (79.8%)	128 (78.5%)	121 (81.2%)

Values reported as n (%), (n) mean ± SD (min–max)

*Acute kidney injury: KDIGO Stage 1: ≥ 26.52 µmol/L increase within 48 h or 1.5–1.9 times baseline within 7 days; Stage 2: 2.0–2.9 times baseline within 7 days; Stage 3: ≥ 3 times baseline within 7 days or increase to ≥ 353.6 µmol/L with an acute increase of > 44.2 µmol/L

Chronic kidney disease: Defined in comorbidities as moderate renal disease: creatinine clearance 51–85 mL/min; and severe renal disease: creatinine clearance less than 50 mL/min and not on dialysis

### Protein and energy delivery in AKI patients

**Fig. 2:**
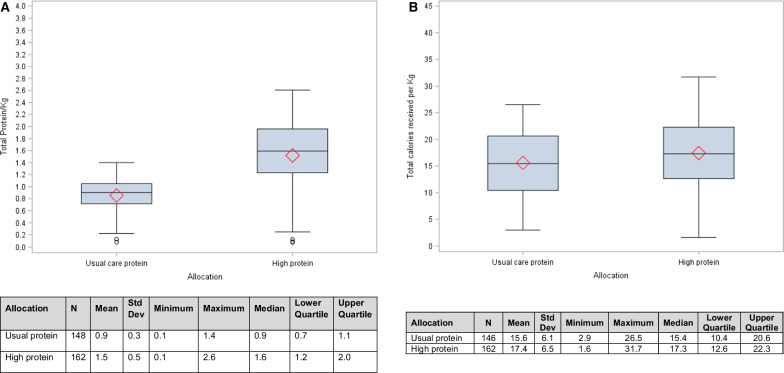
**A** Average daily amounts of protein received in AKI patients (g/kg/body weight protein, up to 28 days from randomization). **B** Average daily amounts of energy received in AKI patients (kcal/kg, up to 12 days from randomization)

Patients in the high and usual protein groups received 1.5 ± 0.5 and 0.9 ± 0.3 g/kg/BW/d of protein during the follow up of 28 days after randomization (Fig. 2A). The high protein group, compared to usual protein group, received slightly higher energy (17.4 ± 6.5 vs. 15.6 ± 6.1 kcal/kg/BW/d; *p* = 0.01) over the first 12 days after randomization (Fig. 2B). Daily amounts of protein and energy received by each group after randomization are shown in Additional file 1: Figs. S1 and S2).

#### Association of protein dose with duration and incidence of kidney replacement therapy

In patients receiving KRT, high or usual protein dosing did not significantly affect the duration of KRT post randomization (5.0 [2.0–10.0] vs. 6.0 [4.0–10.0] days; *p* = 0.21). The overall incidence of new KRT after randomization was 64 (20.5%), and this was not different between the two groups (34 [20.9%] vs. 30 [20.1%]; *p* = 0.87) (Table 2).

**Table 2:** Incidence and duration of new kidney replacement therapy and urea levels

W	AKI patients	High protein	Usual protein	*p* values
	(*n* = 312)	(*n* = 163)	(*n* = 149)	
Duration of kidney replacement from randomization (among patients who received dialysis)* *Median [IQR]*	(107) 6.0 [3.0–10.0]	(60) 5.0 [2.0–10.0]	(47) 6.0 [4.0–10.0]	0.21
New KRT after randomization	64 (20.5%)	34 (20.9%)	30 (20.1%)	0.87
Average Urea (all patients) (mmol/L)	18.7 ± 9.8	19.7 ± 9.8	17.6 ± 9.7	0.04
Average urea ≥ 30 mmol/L (all patients)	42 (13.5%)	26 (16.0%)	16 (10.9%)	0.18

KRT, Kidney replacement therapy

*The duration of KRT from randomization includes 64 incident and 43 prevalent cases of KRT

#### Time course of urea level

Over the observation period, the average levels of urea were higher in the AKI patients receiving high protein dose, irrespective of the use of KRT, when compared to patients with usual dose of protein (19.7 ± 9.8 vs. 17.6 ± 9.7; *p* = 0.04) (Table 2); this difference would not be statistically significant by any reasonable adjustment or multiplicity of testing. The serum levels of urea measured over the time course of 12 days, indicated higher levels of urea in AKI patients that received high protein (arm by time interaction: *p* = 0.02) (Additional file 1: Fig. S3, Table S1).

### Association of protein dose on time-to-discharge-alive

**Fig. 3:**
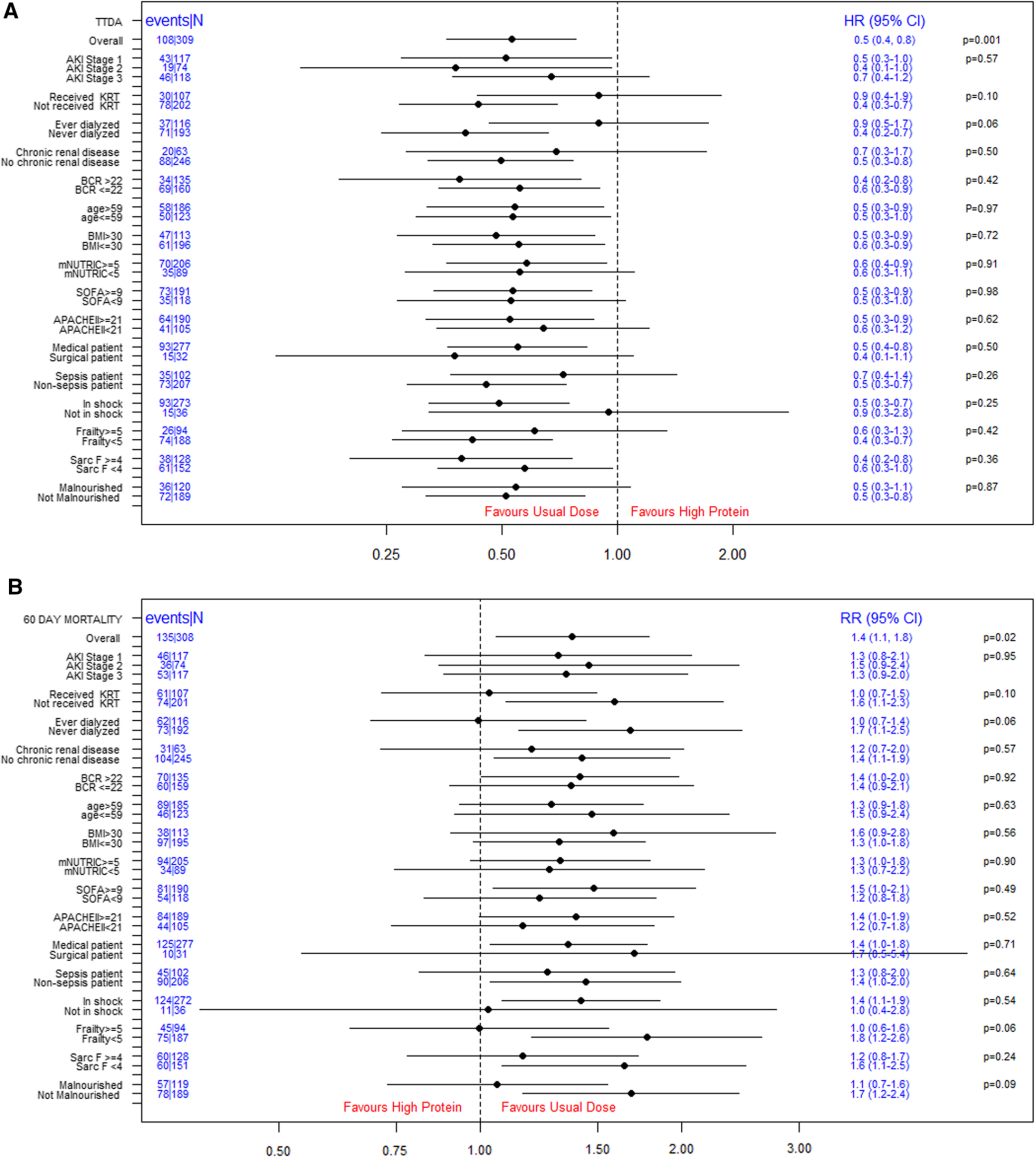
**A** Subgroup analysis for TTDA in patients with AKI. **B** Subgroup analysis for 60-day mortality in patients with AKI. *p*-value: interaction between the subgroups. AKI: Acute kidney injury, APACHE II score: the acute physiology and chronic health evaluation II score, BMI: body mass index, CI: confidence interval, Frailty: measured using the clinical frailty scale, HR: Hazard ratio, KRT: kidney replacement therapy, mNUTRIC: the nutrition risk in critically ill score, SARC-F: a questionnaire to measure risk of sarcopenia, SOFA: sequential organ failure assessment, TTDA: time-to-discharge alive from the hospital. *Note*: Received versus not received KRT—patients who received or not received KRT post randomization. Ever versus never dialyzed—patients who ever or never dialyzed prior to or during the current ICU stay

Amongst all patients with AKI, a higher protein dose, compared to usual, is associated with a slower TTDA (HR 0.5, 95% CI 0.4–0.8; *p* = 0.001 Fig. 3A). The signal of slower TTDA with higher protein dosing is further consistent across all subgroup analysis, including the different AKI stages 1–3 (Fig. 3A).

It is intriguing that the negative effect of higher protein appeared to not be present in patients who were ever dialyzed or received KRT post randomization. However, it should be noted that the corresponding tests for interaction were not statistically significant even before accounting for multiplicity of tests. TTDA was slower across all other subgroups with no significant tests of interaction (Fig. 3A).

### Association of protein dose on mortality

Higher protein dose, compared to usual, was associated with higher 60-day mortality (RR 1.4, 95% CI 1.1–1.8; *p* = 0.02; Fig. 3B) and this effect was similar across AKI stage.

None of the subgroup by protein interactions were statistically significant and no subgroup had lower mortality in the higher protein group (Fig. 3B). However, consistent with TTDA, the observed higher mortality in the higher protein group was limited to patients who did not receive KRT or were never dialyzed.

## Discussion

In this secondary analysis of the largest RCT comparing protein dose in mechanically ventilated critically ill patients, higher protein dose, as compared to usual dose, in patients with AKI was associated with slower TTDA and increased 60-day mortality regardless of the stages of AKI. Interestingly, this harm did not persist in patients who received KRT, albeit the interaction was not statistically significant. High-dose protein was not associated with longer duration or higher incidence of KRT.

Critically ill patients who develop AKI are at risk of protein–energy malnutrition, which is a major negative prognostic factor. Severe malnutrition has been documented in up to 40% of critically ill patients, which is associated with a further increase in morbidity and mortality [19–23]. Therefore, adequate energy and protein intake seems to be crucial component of ICU patient care to prevent deterioration of the nutritional status and its complications. A previous RCT demonstrated that patients with AKI and KRT who received up to 2.5 g/kg/d protein achieved positive nitrogen balance and had improved clinical outcomes, compared to those patients who received a lower dose [24]. Yet, this preliminary evidence was from a small single centre study only, which significantly limits the generalizability of the received findings. Therefore, the optimal nutritional support in AKI still remains an open issue and high-quality evidence in support of this hypothesis are still missing [5]. In this context, the present findings represent the largest analysis to-date, which highlights that higher protein dose, compared to usual dose, in critically patients with AKI is associated with slower TTDA and increased mortality. It is important to mention that the current findings are consistent with findings of previous analyses, which demonstrated that patients with acute kidney injury, were harmed by an additional administration of amino acids [25, 26]. A more careful approach of protein dosing in AKI patients seems warranted in the clinical practice.

There are potential explanations for our findings. First, experimental conditions and theoretical considerations differ from real-world clinical scenarios [27]. It has been previously shown that AKI in critically ill patients is not, per se, associated with increased protein catabolism. Even when present, the underlying mechanisms of protein catabolism are multifactorial and the catabolic state cannot be simply countered by the provision of a higher dose of proteins. The plasma and intracellular amino acid pool have been shown to be altered in critically ill patients with AKI and tissue amino acid utilization is impaired and transport into muscle is reduced, which may partially be attributed to metabolic acidosis [28–31]. The higher urea levels observed in AKI patients, needs to be interpreted carefully within the limitations of an explorative analysis. Further mechanistic and translational studies are however encouraged to test, if the higher urea levels in the high protein group indicate a biological signal of harm, resulting from a reduced capacity to utilize the amino acids during critical illness. These impairments thus may limit the efficacy of delivered nutrition, particularly in preserving muscle mass in critically ill patients. Therefore, exogenously administered protein may increase metabolic stress.

Second, while the duration of dialysis and incidence of new dialysis were not significantly affected by the administration of higher proteins, KRT may have reduced a negative effect of higher proteins on outcomes in patients with AKI. KRT trigger a loss of amino acids, small proteins/peptides up to 5–22 g/day and other nutritional losses such as trace elements and vitamins [7, 32]. Due to its low molecular weight, amino acids such as cysteine, arginine, alanine, and glutamine can be readily filtered from the blood into effluent [7]. During continuous KRT, daily loss of almost 20 g of protein per day is reported [7, 32]. This removal of the excessive protein from the high protein group may explain why we found that higher protein had no effect on TTDA in patients with AKI who received dialysis. In this context, it must be acknowledged that rules for starting and discontinuing KRT are known to significantly vary between institutions and often not standardized across sites.

Third, based on expert consensus, current guidelines postulate that protein requirements are significantly increased in critically ill patients, including those with AKI [24, 32, 33]. Yet, our data could not confirm the benefits of delivering a high protein dose. In accordance with previous findings, it may be possible that the cohort of critically ill patients may have been too sick to benefit from high dose protein [34]. Despite relatively normal protein digestion and amino acid absorption in critically ill patients, the capacity to utilize the protein delivered may be blunted in critically ill patients [31], and high protein intake is unable to reverse this catabolic state [32, 33]. As multiple large RCTs repeatedly failed to demonstrate clinical benefits of nutritional interventions in a heterogenous population of critically ill patients, surrogate biologic markers may help to identify patients that may benefit from a nutritional intervention [35].

In hospitalized patients with AKI not receiving KRT, current nutrition guidelines suggest a range of 1.0–2.0 g/kg/BW/d of protein [10]. While for critically ill patients with AKI receiving KRT, guidelines recommend up to 2.5 g/kg/BW/d [11]. Our findings indicate that mechanically ventilated patients with AKI are harmed by higher protein dosing, particularly in patients who did not receive KRT, so that a careful re-evaluation of the current guideline recommendations for patients with AKI seems warranted.

Our secondary analysis has several strengths. First, we utilized data from the largest (to date) protein dose RCT in critically ill patients. Second, the data included a diverse sample from multiple practice settings worldwide, all of which enhance the generalizability of the received findings. Third, we identified a hypothesis-generating signal to inform practice and future trial design. We acknowledge several limitations of this exploratory secondary analysis. First, the modality of KRT could impact protein requirements, which was not evaluated in this study that included data from a pragmatic design. Second, post-hoc analysis of RCTs needs to be interpreted cautiously and should be considered hypothesis generating, precluding strong clinical recommendation. Third, as AKI was mainly diagnosed from randomization, we are unable to be certain whether the higher protein dose leads to AKI and worsened clinical outcome, or the continuous high protein dose after AKI is developed worsened clinical outcomes, but we note that the rate of AKI was similar in both protein groups so the later explanation seems more plausible. Nevertheless, we must interpret within subgroup treatment effects cautiously for AKI and all subgroups that were defined based on post-randomization data. Finally, there is a risk of type I errors since several subgroups were tested without adjustment for multiplicity, and conversely there is a risk of type II errors since the study may not be adequately powered for the interaction tests used to determine the significance of subgroup effects. Nevertheless, the overall signal of harm in critically ill patients with AKI cannot be ignored and should be considered in nutrition guidelines and future trial designs.

## Conclusion

Higher protein dose, compared to usual dose, is associated with worse clinical outcomes in mechanically ventilated, critically ill patients with AKI. No effect of either higher or lower protein dosing was observed in patients with AKI who received KRT. Based on these findings, current guidelines for nutrition support in critically ill patients with AKI should be carefully re-evaluated and further research is warranted.

### Supplementary Information

**Additional file 1: Figure S1** Protein received in the first 28 days after randomization. **Figure S2** Energy received for the first 12 days after randomization. **Figure S3** Comparison of Urea levels between the treatment groups during the observation period. **Table S1** Urea Levels (mmol/L) over Study Days by Treatment Arms.

## Data Availability

Data collected for the underlying EFFORT protein study will not be publicly available but are being used internally for secondary purposes. Data dictionary or other study tools are available from the coordinating authors (DKH and CS) upon request.

## Abbreviations

AKI: Acute kidney injury
BMI: Body mass index
BUN: Blood urea nitrogen
BW: Body weight
CKD: Chronic kidney disease
ICU: Intensive care unit
KDIGO: Kidney disease improving global outcomes
KRT: Kidney replacement therapy
TTDA: Time to discharge alive from hospital
