# Supplementary material for: The impact of higher protein dosing on outcomes in critically ill patients with acute kidney injury: a post hoc analysis of the EFFORT protein trial
Source: Crit Care. 2023 Oct 18;27:399. doi: 10.1186/s13054-023-04663-8 (PMC10585921; doi:10.1186/s13054-023-04663-8)
Supplement: Supplementary file 1 — Additional file 1: Figure S1 Protein received in the first 28 days after randomization. Figure S2 Energy received for the first 12 days after randomization. Figure S3 Comparison of Urea levels between the treatment groups during the observation period. Table S1 Urea Levels (mmol/L) over Study Days by Treatment Arms. [file 13054_2023_4663_MOESM1_ESM.docx]

**Supplementary Appendix**

**Supplemental Figure 1**

**Protein received in the first 28 days after randomization**

**
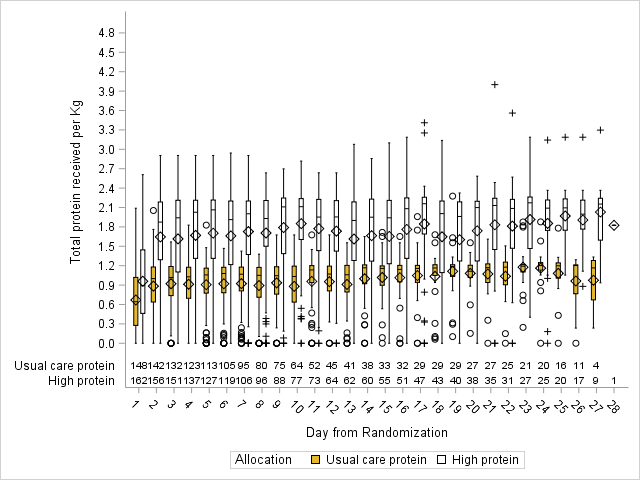
**

**Supplemental Figure 2**

**Energy received for the first 12 days after randomization**

**
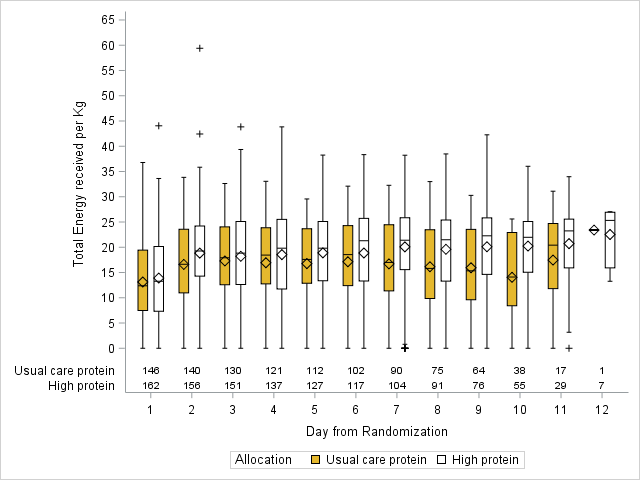
**

**Supplemental Figure 3**

**Comparison of Urea levels between the treatment groups during the observation period**


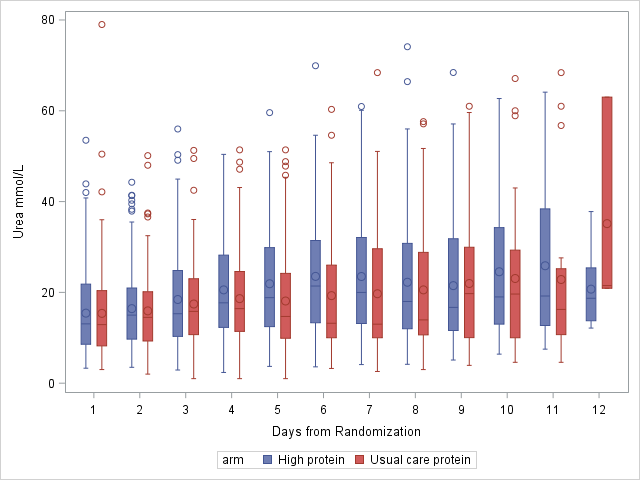


**Supplemental Table 1 - Urea Levels (mmol/L) over Study Days by Treatment Arms**

| **Day #** | **1** | **2** | **3** | **4** | **5** | **6** | **7** | **8** | **9** | **10** | **11** | **12** |
| --- | --- | --- | --- | --- | --- | --- | --- | --- | --- | --- | --- | --- |
| **High Protein** | (142) 15.4±15.4 | (139) 15.9±15.9 | (129) 17.4±17.4 | (119) 18.6±18.6 | (115) 18.1±18.1 | (97) 19.3±19.3 | (90) 19.7±19.7 | (76) 20.5±20.5 | (56) 22±22 | (37) 23.1±23.1 | (18) 22.8±22.8 | (3) 35.1±35.1 |
| **Usual Care** | (156) 15.4±15.4 | (153) 16.4±16.4 | (150) 18.4±18.4 | (131) 20.5±20.5 | (120) 21.9±21.9 | (106) 23.5±23.5 | (99) 23.5±23.5 | (87) 22.2±22.2 | (69) 21.5±21.5 | (55) 24.5±24.5 | (27) 25.9±25.9 | (8) 20.7±20.7 |

(n) mean±sd

|  | p values |
| --- | --- |
| Arm (Group) | 0.82 |
| Days after randomization (Time) | <0.0001 |
| Interaction (Group*Time) | 0.02 |
